# Combined Biceps Anterior Cable Reconstruction and Biological Tuberoplasty for Irreparable Rotator Cuff Tears Using a Composite Collagen Matrix–Achilles Tendon Allograft

**DOI:** 10.1016/j.eats.2025.103641

**Published:** 2025-05-22

**Authors:** Kun-Hui Chen, Julius Albert Rabang Yen, En-Rung Chiang, Hsuan-Hsiao Ma, Hsin-Yi Wang, Hsiao-Li Ma

**Affiliations:** a Department of Orthopaedics and Traumatology, Taipei Veterans General Hospital, Taipei, Taiwan; b Department of Surgery, School of Medicine, National Yang Ming Chiao Tung University, Taipei, Taiwan; c Institute of Clinical Medicine, National Yang Ming Chiao Tung University, Taipei, Taiwan; d Allied Care Experts Medical Center, Cebu, Philippines; e Cebu Institute of Medicine, Cebu, Philippines; f Department of Anaesthesiology, Taipei Veterans General Hospital, Taipei, Taiwan

## Abstract

This article describes a technique that combines biceps anterior cable reconstruction with biological tuberoplasty using a composite collagen matrix–Achilles tendon allograft for patients with irreparable rotator cuff tears. These patients often experience loss of the force couple, leading to superior humeral migration, which causes an abutment of the greater tuberosity against the acromion during active deltoid contraction. In addressing this issue, both biceps anterior cable reconstruction and biological tuberoplasty have proved effective. The combined approach offers biomechanical advantages by stabilizing the humeral head through biceps anterior cable reconstruction while biological tuberoplasty provides cushioning and prevents bone-to-bone contact between the acromion and greater tuberosity. We believe these 2 procedures have a synergistic effect, promoting joint preservation, postoperative pain relief, and improved shoulder function.

Irreparable rotator cuff tears (IRRCTs) present significant challenges for surgeons. Many treatment options have been reported.^1^, ^2^, ^3^ Treatment depends on patient-specific factors, rotator cuff conditions, and surgeon preference. IRRCTs often disrupt the force couple mechanism, causing superior migration of the humeral head and resulting in subacromial impingement, which leads to pain and reduced shoulder function.

This article presents a combined surgical approach using biceps anterior cable reconstruction (ACR), partial rotator cuff repair, and biological tuberoplasty (BT) with a composite collagen matrix–Achilles tendon allograft (CCMAT). This technique aims to stabilize the humeral head, reduce impingement, decrease postoperative pain, and improve shoulder function.

## Surgical Technique

### Patient Position

**Fig 1:**
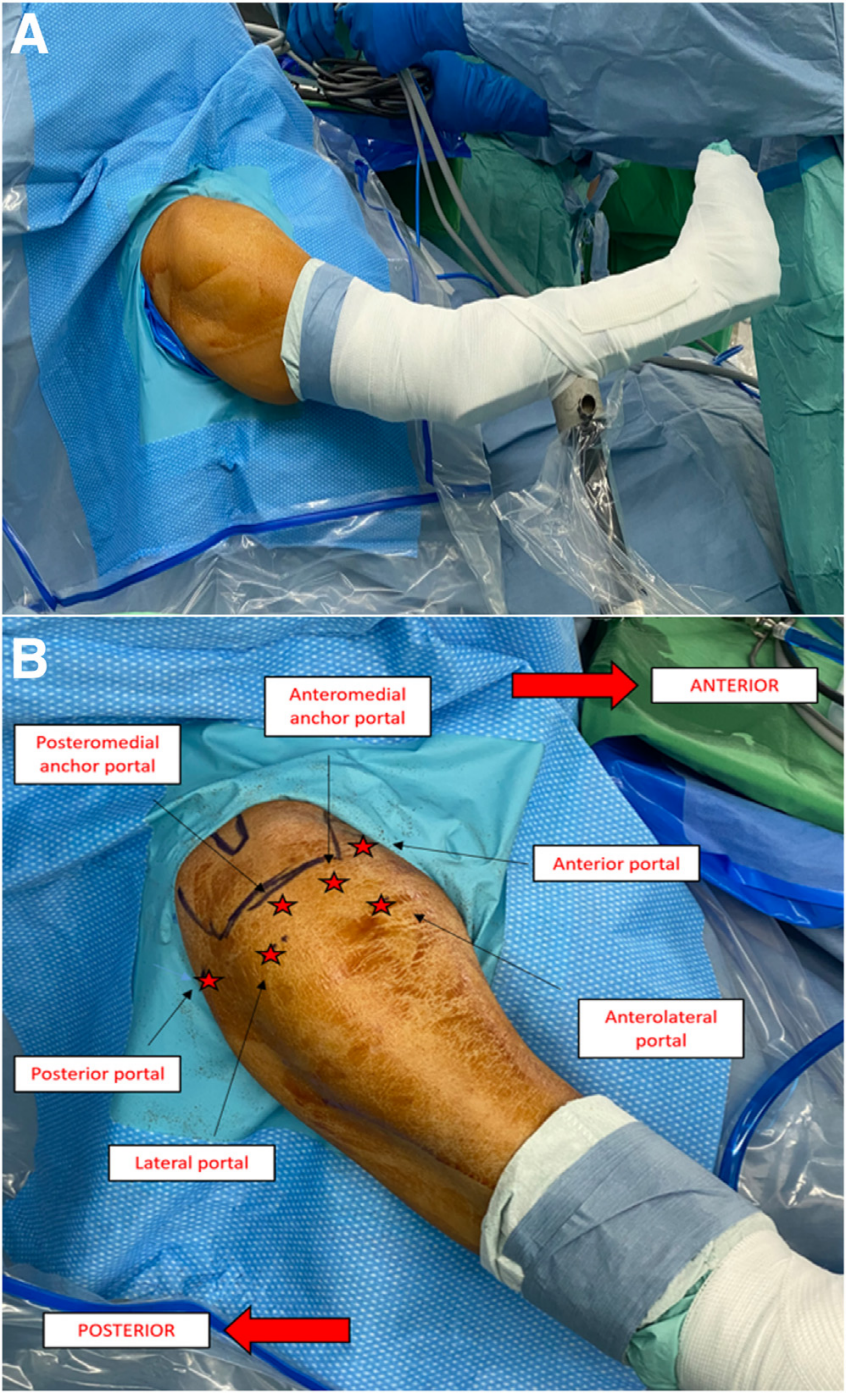
(A) Arm positioning of a right arm with the patient in the beach-chair position. (B) Portals (stars) for surgery.

The patient is placed in the beach-chair position under intravenous general anesthesia with an interscalene block (Video 1). The shoulder is then prepared aseptically and draped to expose the operative area with an arm holder (Fig 1A).

### Diagnostic Arthroscopy

The portal sites are identified using osseous landmarks (Fig 1B). Through the posterior portal, the posterosuperior cuff tear, intact subscapular tendon, and biceps tendon are identified. The anterior, anterolateral, and lateral portals are created. The viewing portal is then changed to the lateral portal. The anterolateral portal serves as the primary operative portal.

### Rotator Cuff Release and Evaluation of Irreparability of Supraspinatus Tendon

**Fig 2:**
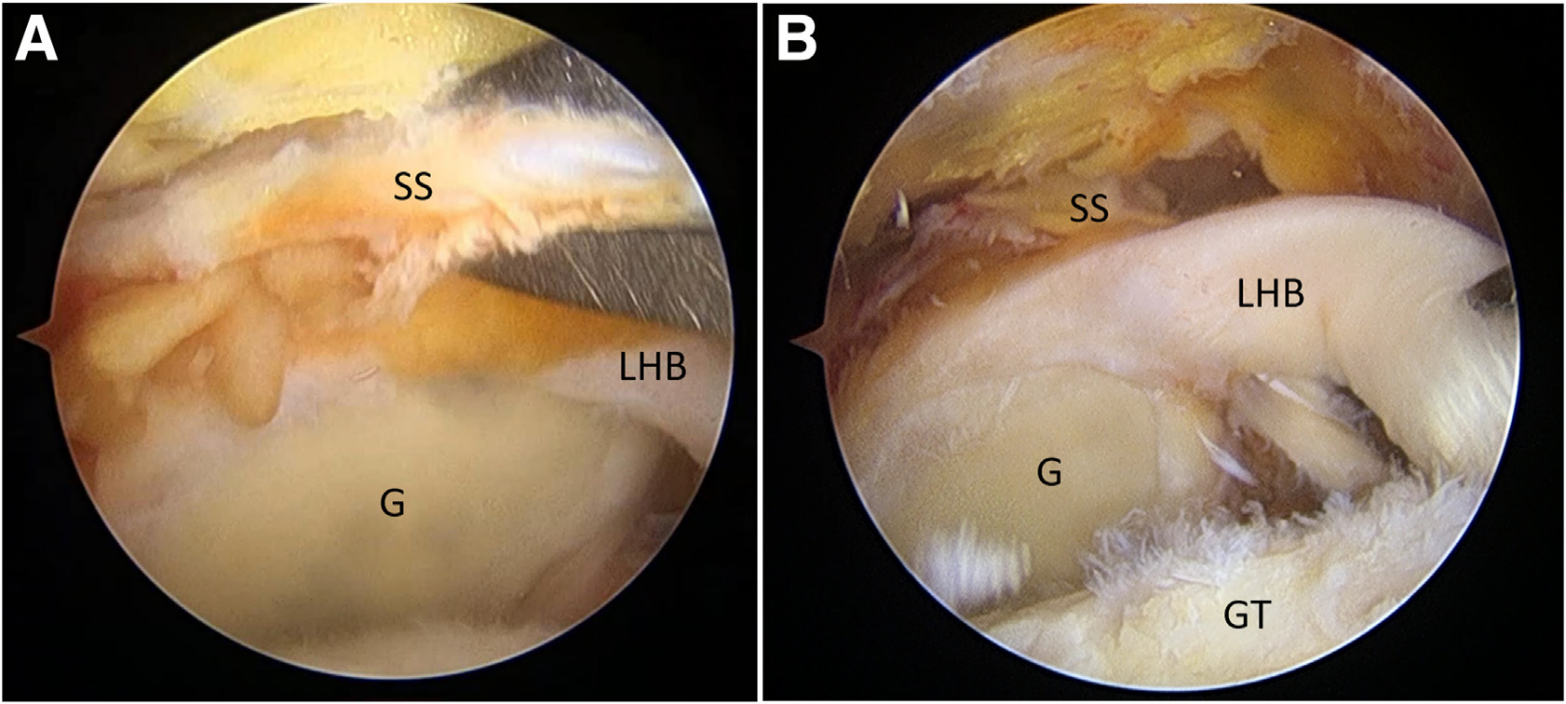
Irreparable supraspinatus tendon (SS) and intact long head of biceps tendon (LHB), viewing from lateral portal. (A) A Grasping Froceps is used to pull the SS, which cannot reach the greater tuberosity (GT). (B) The LHB is healthy and without any tears. (G, glenoid.)

Adequate release of the rotator cuff is subsequently performed, including release of the coracohumeral ligament. If the surgeon is unable to reach the medial edge by pulling laterally even after medialization of the greater tuberosity (GT) and the biceps is intact, the combined biceps ACR and BT approach is used (Fig 2).

### Biceps ACR

**Fig 3:**
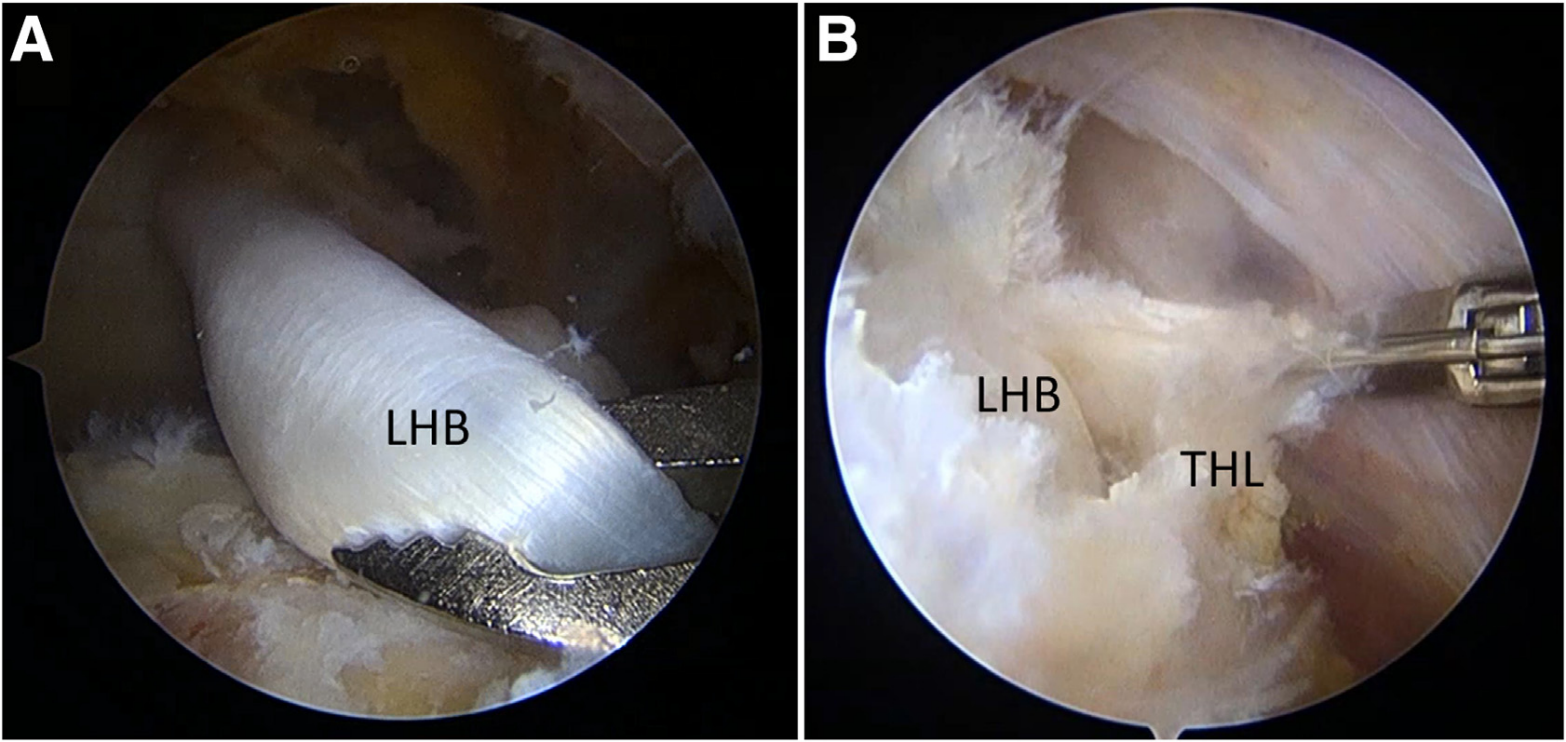
Assessment of long head of biceps tendon (LHB) mobility and release of transverse humeral ligament (THL) in right shoulder, viewing from lateral portal. (A) The LHB is pulled posteriorly to assess its mobility to reach the position of the anterior cable. (B) If the tension is too high, the THL is released.

**Fig 4:**
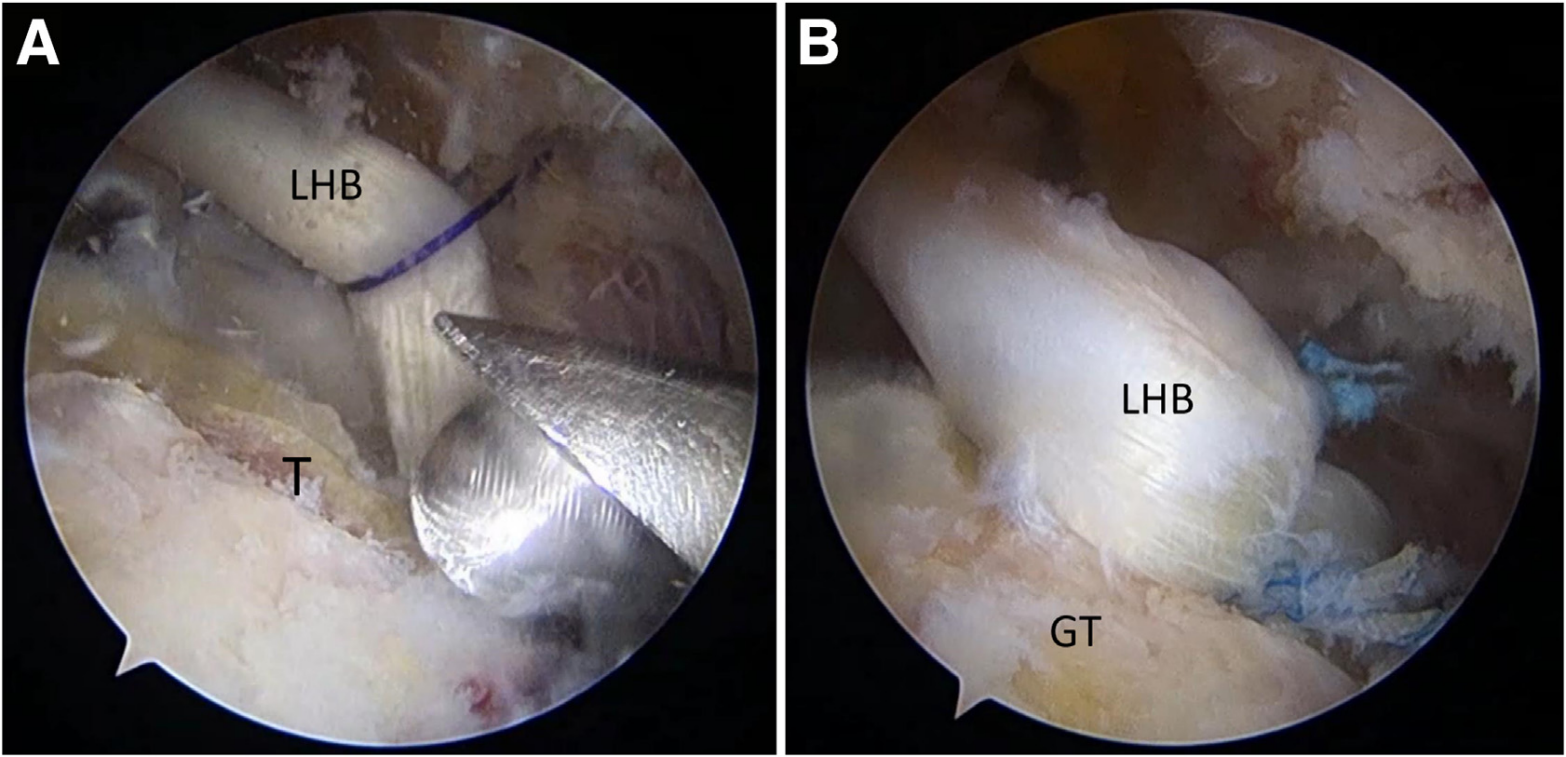
Creation of bone trough and completion of distal portion of biceps ABR, viewing from lateral portal. (A) Trough creation. (B) The distal portion of the biceps ACR is fixed with a suture anchor. (GT, greater tuberosity, LHB, long head of biceps tendon; T, trough for ACR.)

Through the working portal, the biceps tendon is pulled posteriorly to assess its mobility. If it is deemed insufficient, the transverse humeral ligament is released to provide more mobility (Fig 3). Next, a bone trough is created using an arthroscopic burr approximately 10 mm posterior to the bicipital groove, where the pulled biceps tendon would lie. An anteromedial anchor portal is created for anchor placement onto the bone trough. A suture anchor is then placed at the lateral section of the trough, and the biceps tendon is fixed by this anchor. Distal fixation of the ACR is therefore completed (Fig 4).

### Tuberosity Footprint Preparation, Medial Anchor Placement, Biceps Fixation, and Infraspinatus Tendon Repair

**Fig 5:**
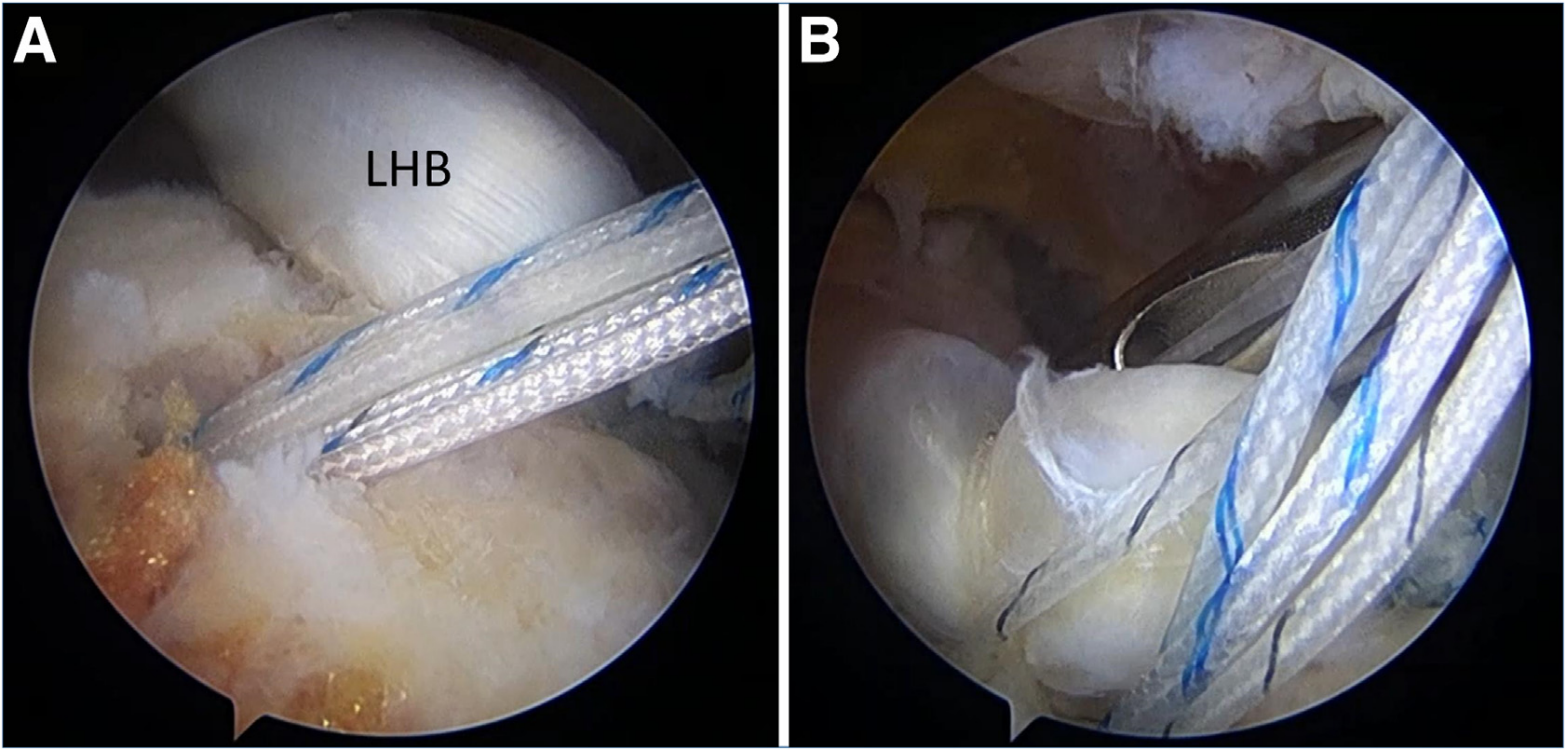
Anteromedial anchor and medial part of rerouted biceps tendon fixation, viewing from lateral portal. (A) A triple-loaded suture anchor is placed on the anteromedial portion of the greater tuberosity, posterior to the medial part of the rerouted biceps tendon. (B) One strand of the suture is used to fix the medial part of the rerouted biceps tendon. (LHB, long head of biceps tendon.)

**Fig 6:**
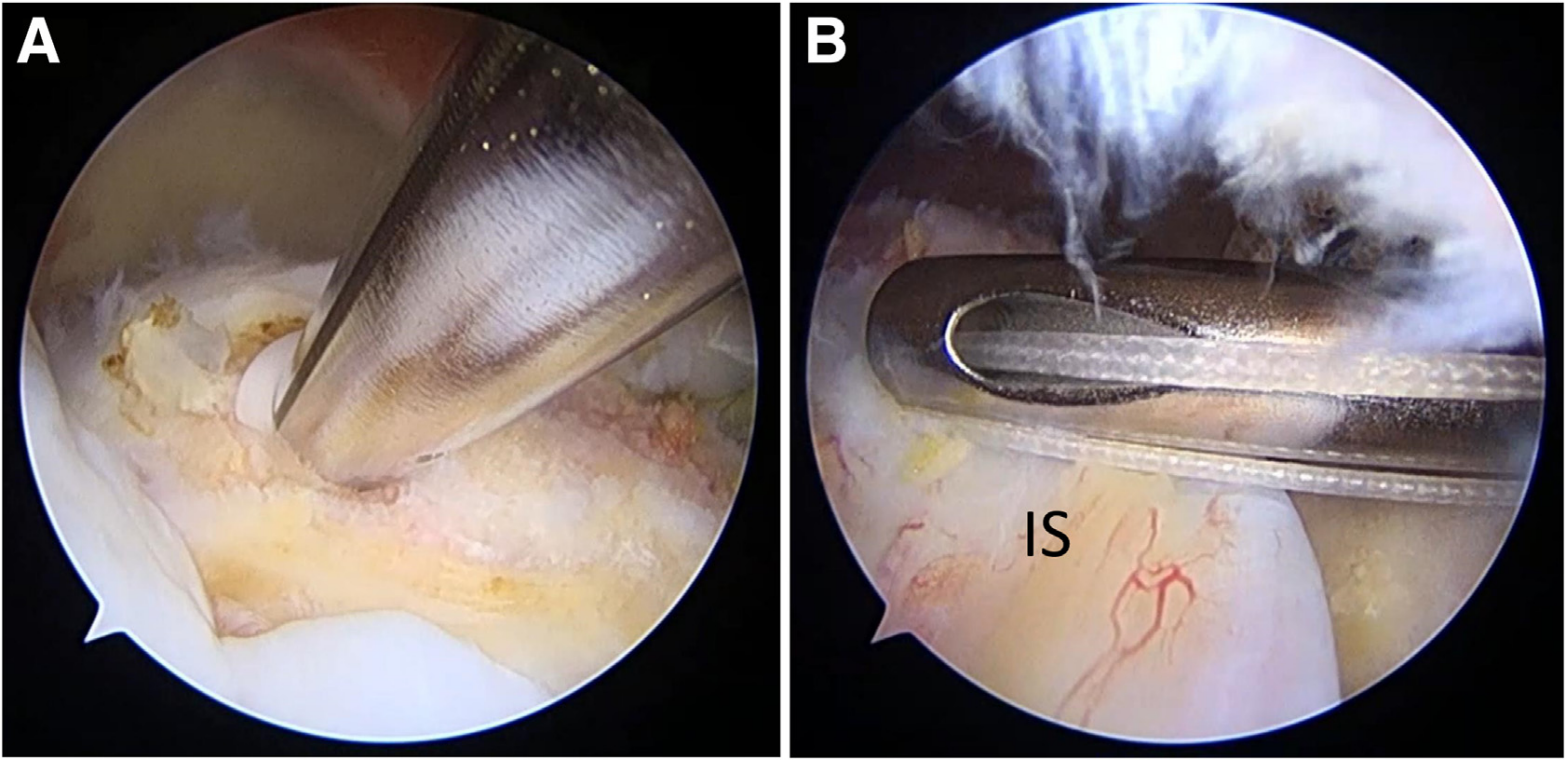
Posteromedial anchor placement and infraspinatus tendon (IS) repair, viewing from lateral portal. (A) A triple-loaded suture anchor is placed on the posteromedial portion of the greater tuberosity, just anterior to the IS, for low-tension repair. (B) One strand of the suture is used to repair the IS.

Next, the footprint is prepared by slight decortication until slight bleeding occurs in the GT. By use of the anteromedial anchor portal, a triple-loaded suture anchor (Genesys; ConMed) is placed on the anteromedial portion of the GT, which is posterior to the medial part of the rerouted biceps tendon. For easy distinction of the strands, they are labeled as strands 1, 2, and 3. Strand 1 from this anchor is used to fix the medial part of the rerouted biceps tendon (Fig 5). A posteromedial anchor portal is used to place another anchor on the posteromedial aspect of the GT. Then, strand 1 from this anchor (Fig 6) is used to perform the infraspinatus (IS) repair. These steps include the ACR and repair of the IS. The remaining bare area of the GT is measured for graft size determination.

### Graft Preparation and Shuttling

**Fig 7:**
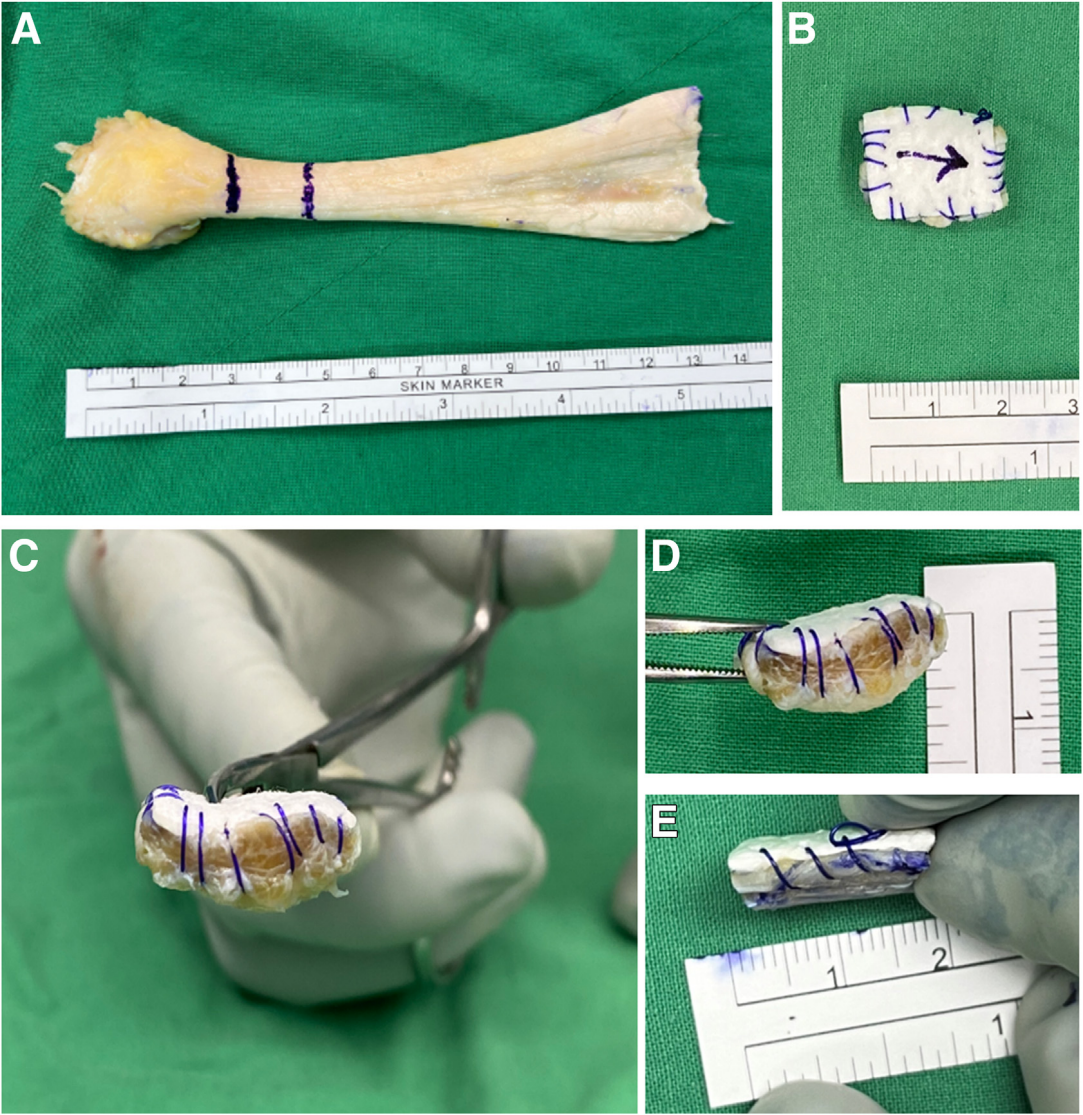
Composite graft preparation. (A) A 1.5 × 1.5-cm Achilles tendon allograft is prepared and cut from the calcaneal insertion to approximately 1.5 cm proximally to the allograft. (B) The collagen matrix is placed on top of the Achilles allograft, and the collagen matrix and allograft are sutured together in a running suture technique on all sides. The arrow, indicating the top position intra-articularly, should be noted. (C-E) The graft thickness can be up to 8 mm.

On the working table, in this case, a 1.5 × 1.5-cm Achilles tendon allograft is cut from the calcaneal insertion. A collagen matrix (ABCcolla; ACRO Biomedical) is cut to the same size as the Achilles tendon allograft. The piece is then placed on top of the Achilles tendon allograft, and the collagen matrix and allograft are sutured together on all sides of the composite graft using a running suture technique. The top of the composite graft is marked with an arrow to facilitate visualization inside the joint (Fig 7).

One limb of strand 2 from the anteromedial anchor is passed under the fixed biceps tendon, positioned medially near the humeral articular edge, and retrieved through the anterior portal. Subsequently, 1 limb of strand 2 from the posteromedial anchor is passed through the IS and retrieved through the posterior portal. Finally, the limbs of strand 3 from the anteromedial and posteromedial anchors are retrieved through the anterior and posterior portals, respectively, without passing through the biceps or IS tendon.

**Fig 8:**
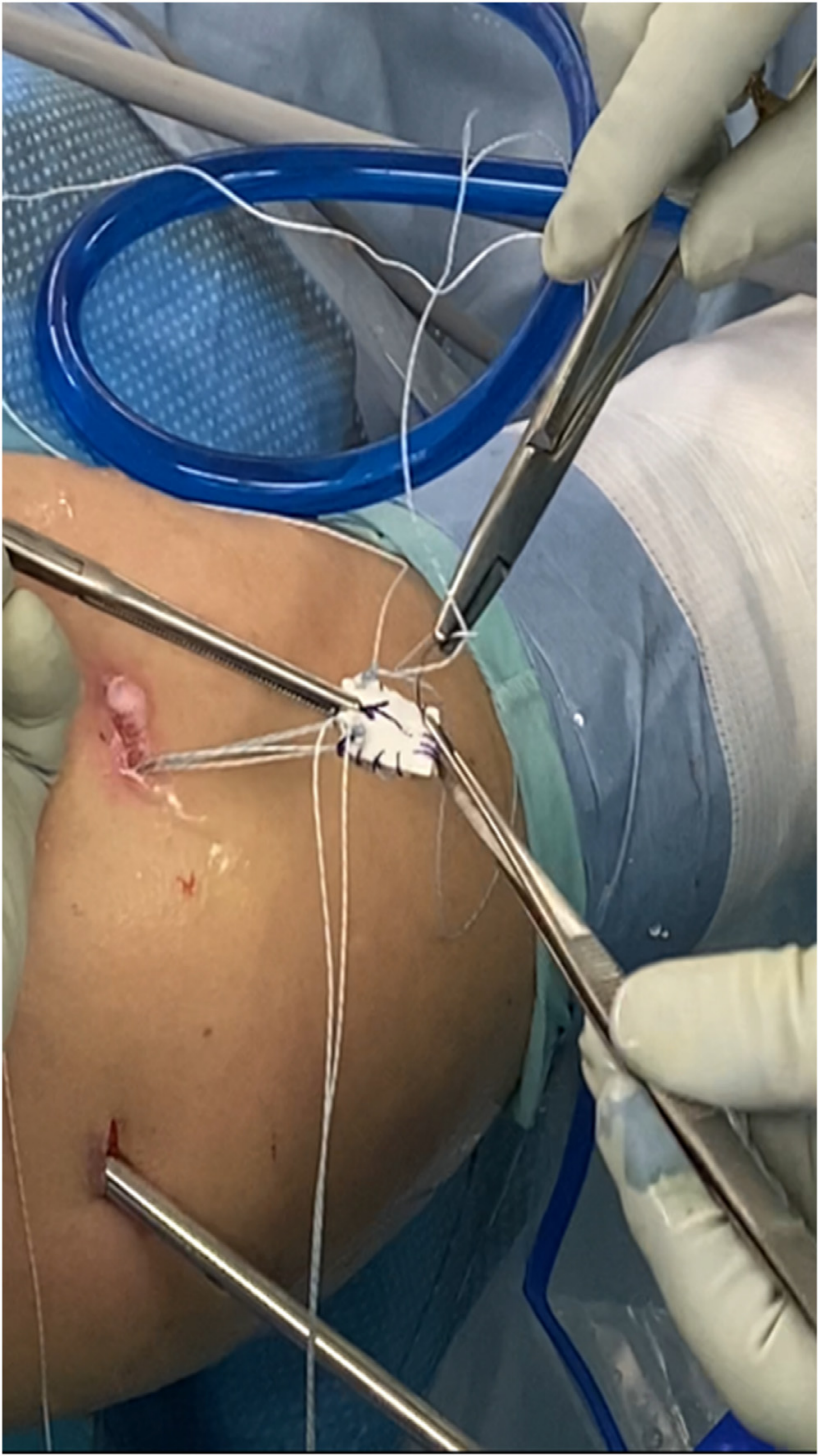
Suture management with composite graft.

**Fig 9:**
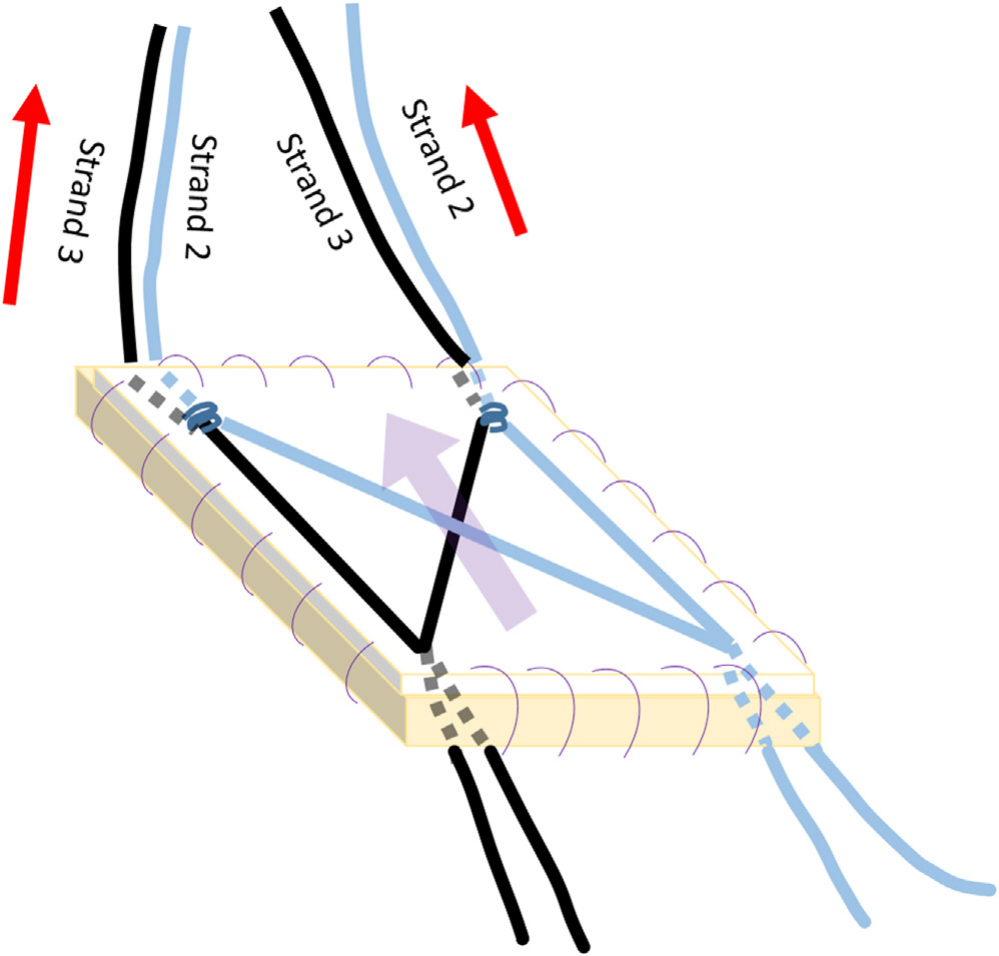
Suture passage through composite collagen matrix–Achilles tendon allograft. The limbs of strands 2 and 3 from the 2 medial anchors are tied to the anteromedial and posteromedial corners of the composite graft.

The remaining limbs of strands 2 and 3 from the anteromedial and posteromedial anchors are retrieved outside the shoulder through the anterolateral portal. The limbs of strands 2 and 3 from the anteromedial anchor suture are then individually passed to the anteromedial corner of the CCMAT and tied. The remaining limbs of strands 2 and 3 from the posteromedial anchor suture are individually passed to the posteromedial corner of the CCMAT and tied. Finally, the remaining limbs of the same color strands from the anteromedial corner and posteromedial corner of the CCMAT are passed to the anterolateral corner and posterolateral corner of the CCMAT, respectively (Figs 8 and 9).

**Fig 10:**
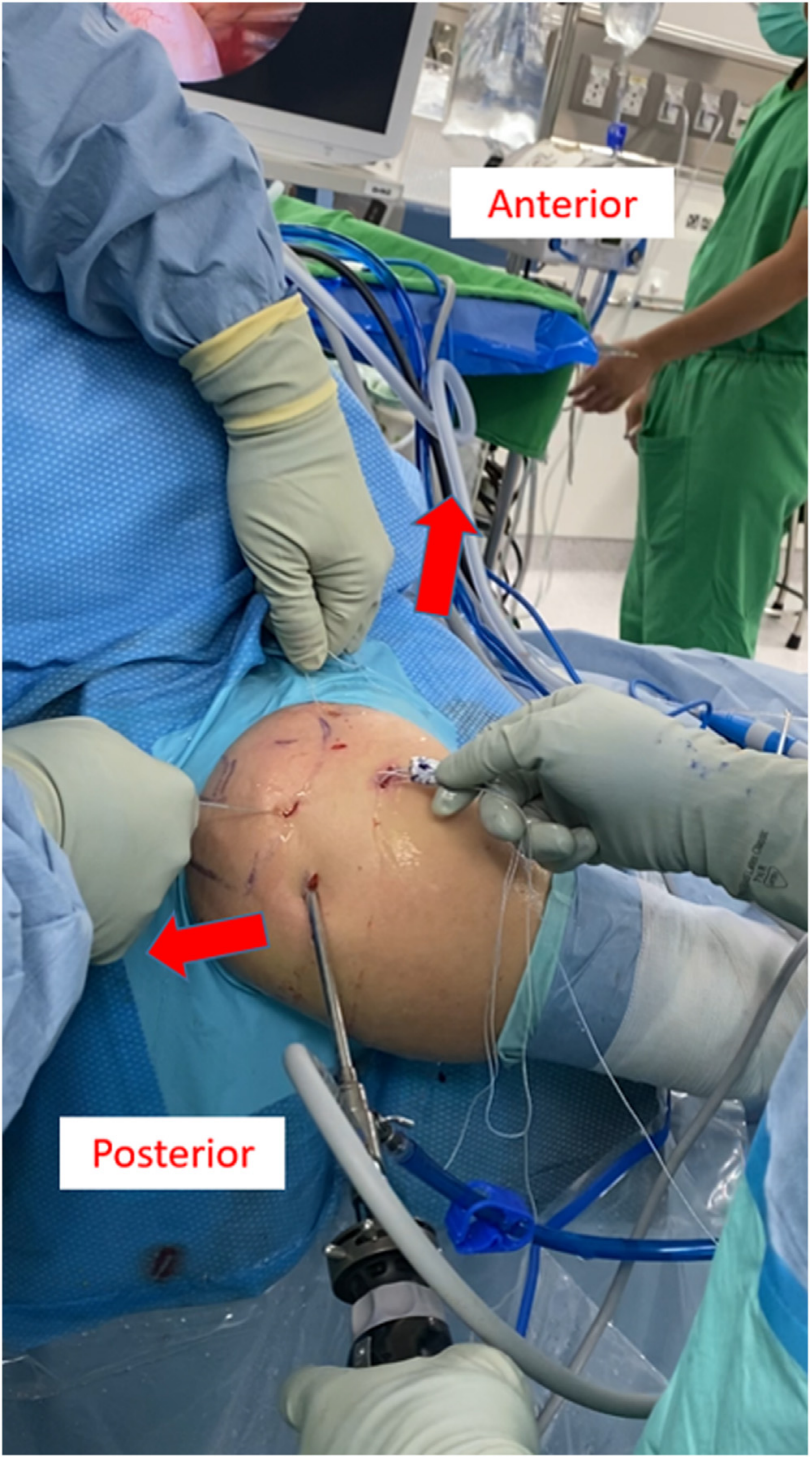
Graft shuttling in joint. The graft is delivered intra-articularly toward the footprint by pulling the proximal limb strands of the anteromedial and posteromedial anchor sutures located in the anterior and posterior portals, respectively.

**Fig 11:**
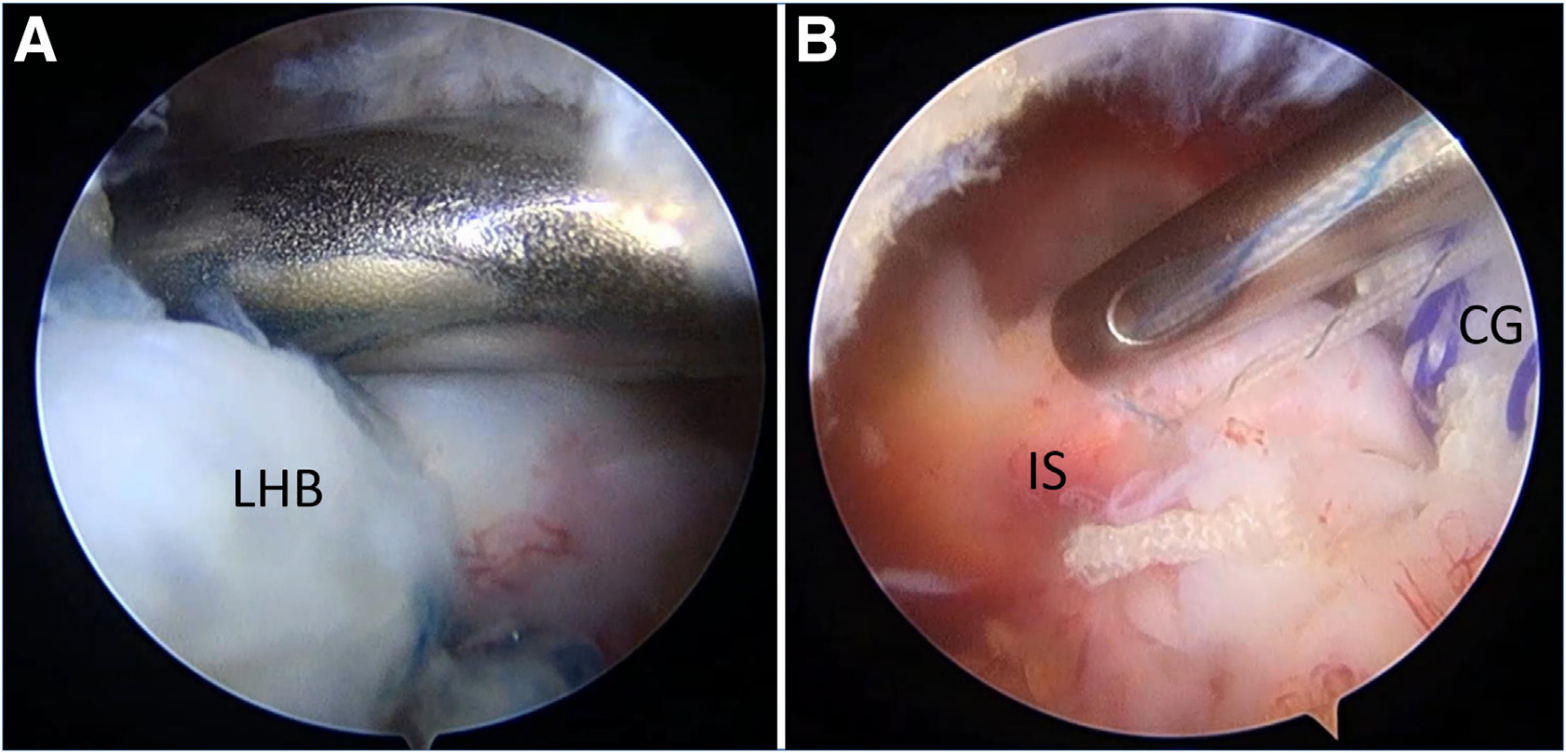
Medial-row composite graft (CG) fixation, viewing from lateral portal. (A) The suture limbs from the anterior portal are tied to fix the anteromedial corner of the CG to the footprint while fixing the proximal part of the rerouted biceps tendon. (B) The suture limbs from the posterior portal are tied to fix the posteromedial corner of the CG to the footprint and repair the infraspinatus tendon (IS) simultaneously. (LHB, long head of biceps tendon.)

**Fig 12:**
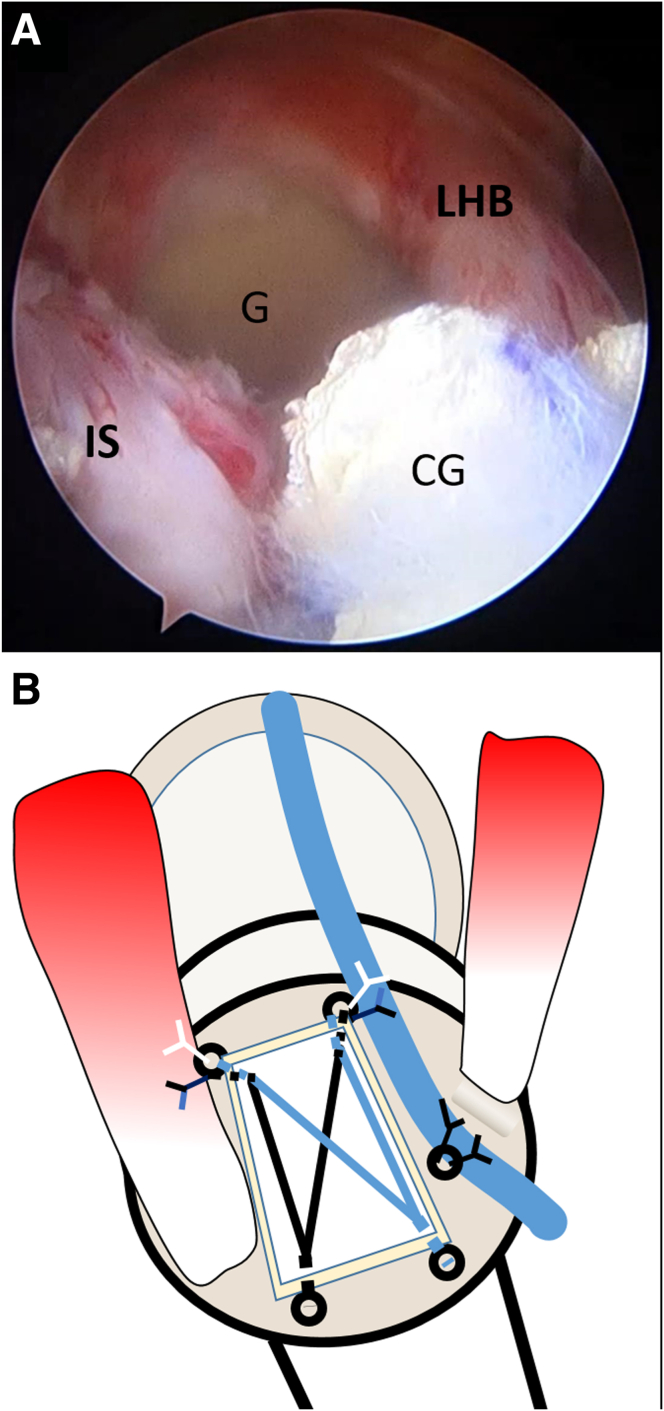
Final construct. Arthroscopic (A) and graphic (B) representations of completed biceps rerouting with anterior cable reconstruction, repair of the infraspinatus toward the footprint of the humerus with low tension, and application of biological tuberoplasty to the remaining bare area of the footprint. (CG, composite graft; G, glenoid; IS, infraspinatus tendon; LHB, long head of biceps tendon.)

The CCMAT is delivered toward the GT by pulling the suture from the anterior and posterior portals, respectively (Fig 10). Once the CCMAT is placed on the bare area of the GT, the suture limbs from the anterior and posterior portals are tied to fix the anteromedial corner of the CCMAT while fixing the proximal part of the rerouted biceps tendon and to fix the posteromedial corner of the CCMAT while simultaneously repairing the IS (Fig 11). Lateral-row fixation is then performed, at which stage the surgical technique is complete. The final construct, comprising a combination of ACR and BT, is shown in Figure 12. The skin incisions are then sutured using No. 3-0 nylon. Table 1 outlines guidelines for the technique and potential pitfalls.

**Table 1:** Technique Guidelines and Potential Pitfalls

Surgical Step	Pearls	Potential Pitfalls
ACR	The position for biceps anterior cable reconstruction should be found; if the tension is too high for the new position of the biceps, transverse humeral ligament release should be performed.	Without releasing the transverse humeral ligament and pulling the biceps tendon too posteriorly, excessive tension will be placed on the biceps tendon.
Anchor choice	Triple-loaded anchors can be used to fix the composite graft, perform ACR, and repair the IS.	Double-loaded anchors cannot be used to simultaneously fix the composite graft, perform biceps fixation, and conduct 2 single-row IS repairs.
Graft preparation	The collagen matrix can help splint the Achilles allograft and prevent acromial impingement.	Without collagen matrix splinting, the Achilles tendon will wrinkle after 4-sided suturing.
	The collagen matrix should be sutured to the Achilles allograft around its 4 sides.	
	The graft should not be oversized compared with the measured footprint size.	An oversized graft could hinder the scope operation.
Graft shuttling	The limbs of strands 2 and 3 from the medial anchors should be tied at the anteromedial and posteromedial corners of the graft, which helps to pull the graft into the subacromial space.	Without a knot, the graft cannot be shuttled into the space.
Graft fixation	After shutting the graft on the greater tuberosity, the sutures used to pull the graft into the subacromial space should be tied first and then fixed the lateral row.	The lateral row should not be fixed first, which would cause the graft to be pulled laterally.

ACR, anterior cable reconstruction; IS, infraspinatus.

## Discussion

This report describes the use of combined biceps ACR and BT for IRRCTs using a CCMAT graft. Indications and contraindications are listed in Table 2. Advantages and limitations are presented in Table 3.

**Table 2:** Indications and Contraindications

Indications
Irreparable supraspinatus tendon
Reparable infraspinatus and subscapularis tendon
Intact LHBT
No cuff arthropathy
Painful and weak shoulder
Contraindications
Active or with previous infection
Severe osteoarthritis of glenohumeral joint (Kellgren-Lawrence grade III-IV)
Irreparable infraspinatus or severe fatty infiltration of infraspinatus
LHBT tear

LHBT, long head of biceps tendon.

**Table 3:** Advantages and Limitations of Technique

Advantages
Biological tuberoplasty
Provides cushion between greater tuberosity and undersurface of acromion
No need for fixation on superior glenoid side, in contrast to superior capsular reconstruction, reducing surgery duration
Collagen patch
Acts as splint for Achilles tendon graft, allowing it to lie flat on tuberosity surface
Helps distribute load of sutures on Achilles tendon graft during preparation
Protects Achilles tendon graft against potential acromial impingement
Graft thickness is increased, resulting in composite thickness of approximately 8 mm
Makes it easier to label and orient graft, including identifying its front, back, top, and bottom segments
Prevents free floating of surface fibers, a common problem with Achilles tendon grafts
Anterior cable reconstruction
Provides biomechanical stability by normalizing superior migration and reducing subacromial pressure
Eliminates need for additional glenoid fixation, lowering risk of complications
Preserves dynamic function of biceps tendon, aiding in humeral head depression and shoulder mechanics
Limitations
Intact biceps tendon required
Reparable subscapularis and infraspinatus tendons required
Achilles tendon allograft required
Collagen matrix required
Triple-loaded anchor required

ACR is one of the available treatments for IRRCTs.^4^, ^5^, ^6^ Considering the anatomic positioning, the native cable attachment is located slightly posterior and lateral to the bicipital groove, optimizing shoulder alignment and function.^7^ With our technique, we aim to reconstruct the anterior cable at its native attachment site, avoiding excessive tension on the biceps tendon, because in cases of IRRCT, this tendon often shows some degree of longitudinal tearing. Previous literature on ACR recommends repairing the IS first, before performing ACR.^8^ However, in some IRRCT cases, the IS may not fully cover the GT with low tension. To avoid high tension repair in either ACR or IS repair, we recommend reconstructing the anterior cable at its native position while performing a low-tension repair of the IS and using BT for the uncovered GT. We do not recommend performing tenotomy of the biceps tendon, considering its dynamic function association with depression of the humeral head.^9^

Superior capsular reconstruction is a well-accepted treatment option for IRRCTs.^10^^,^^11^ Several studies have found that cases of graft failure, whether on the glenoid side or within the graft itself, can still show positive clinical outcomes as long as the graft remains intact over the GT.^10^^,^^12^^,^^13^ These findings suggest that clinical success may be linked to the interpositional effect of the graft between the GT and acromion, indicating that full graft coverage extending to the glenoid might not be essential for achieving acceptable therapeutic outcomes. As a result, techniques known as “biologic tuberoplasty” or “biologic interpositional tuberoplasty” have emerged.^11^^,^^14^^,^^15^ For these reasons, our approach incorporates and adapts the BT technique.

Achilles tendon allografts have been used in many surgical modalities requiring grafting.^16^, ^17^, ^18^, ^19^, ^20^ However, to our knowledge, this technique is the first to use Achilles tendon allograft for BT. We selected the portion of the Achilles tendon near its calcaneal insertion because it is the thickest and strongest, providing an effective spacer function.

When an Achilles tendon graft is prepared, suturing of the 4 edges of the tendon often causes the graft to wrinkle, making it uneven. Additionally, suturing around the graft may disrupt the fiber alignment of the Achilles tendon, thereby increasing the risk of tearing. Furthermore, the surface fibers of the Achilles tendon graft tend to float in a saline solution–filled environment, which cannot be easily addressed with a shaver. This complicates the surgical procedure because floating fibers obstruct the surgical view. Therefore, we recommend placing a collagen patch on the surface of the Achilles tendon graft and suturing them together, which offers several advantages (as outlined in Table 3). In addition to performing ACR and BT during a single procedure, we use triple-loaded anchors in the medial row, which enables additional double fixation of the biceps tendon, reinforcing the ACR strength and repair of the IS by 2 single-row sutures, without the need for additional anchors.

This technique offers a combined approach involving biceps ACR, partial rotator cuff repair, and BT using a CCMAT. It offers a promising alternative for patients with IRRCTs. Early experience suggests that this method can yield favorable clinical outcomes, preserve joint function, and provide significant postoperative pain relief.

## Funding

Support was provided by the National Scientific and Technical Research Council, Taiwan (113-2314-B-075-047).

## Supplementary Data

Video 1 Key steps of surgical procedure. The patient is positioned in the beach-chair position, with the right shoulder prepared for surgery. Initially, the irreparable supraspinatus tendon is assessed, followed by an evaluation of the low-tension position for infraspinatus tendon repair, along with decortication and medialization of the footprint. The focus then shifts to the biceps tendon. The transverse humeral ligament is released, and the mobility of the biceps tendon is checked. A traction suture is placed to protect the biceps while creating a trough, and a distal anchor is inserted to secure the biceps. The low-tension repair position for the infraspinatus tendon is reassessed. Two triple-loaded suture anchors are placed at the anteromedial and posteromedial corners of the footprint. Strand 1 from the anteromedial anchor is used to fix the medial biceps, whereas strand 1 from the posteromedial anchor is used for infraspinatus repair. One limb each from strand 2 and strand 3 from the anteromedial anchor is retrieved through the anterior portal, with 1 limb of strand 2 passing through the biceps tendon. Similarly, 1 limb each from strand 2 and strand 3 from the posteromedial anchor is retrieved through the posterior portal, with 1 limb of strand 2 passing through the infraspinatus tendon. All remaining sutures are then retrieved through the anterolateral portal. The graft is prepared and positioned into the subacromial space over the footprint by pulling the sutures from the anterior and posterior portals, followed by tying the sutures in both portals. The procedure concludes with lateral-row fixation, completing the operation.
